# Editorial: Innovative Analysis Ecosystems for HEP Data

**DOI:** 10.3389/fdata.2021.736105

**Published:** 2021-08-05

**Authors:** Sezen Sekmen, Gian Michele Innocenti, Bo Jayatilaka

**Affiliations:** ^1^ The Center for High Energy Physics, Kyungpook National University, Daegu, South Korea; ^2^ Physics Department, CERN, Geneva, Switzerland; ^3^ Fermi National Accelerator Laboratory, Batavia, IL, United States

**Keywords:** HPC, innovative analysis framework design, software containers, analysis description language, parallelized analysis, big data, fast processing, machine learning

## Abstract

This editorial summarizes the contributions to the Frontiers Research topic “Innovative Analysis Ecosystems for HEP Data”, established under the Big Data and AI in High Energy Physics section and appearing under the Frontiers in Big Data and Frontiers in Artificial Intelligence journals.

High energy physics (HEP) experiments are collecting an unprecedented amount of data. Major upgrades of the current HEP experiments already in progress will further increase the volume and complexity of these data. This new territory of extremely large data is inspiring and challenging physicists to devise new and more advanced analysis techniques, which feature increasingly more elaborate procedures such as machine learning and deep neural networks, to maximize the physics outcome of these studies. These developments, which will have a growing impact on HEP in the coming years, require fast and efficient analysis ecosystems and state-of-the-art hardware infrastructures. Exploiting recent advancements in data science and machine learning has already led to a significant leap in HEP data analysis. It is therefore of high interest to continue in this effect to maximize the impact of these new techniques in HEP.

This research topic aimed to explore recent developments in this area with a focus on 1) innovative, user-friendly and preservable analysis frameworks and dedicated languages describing analysis algorithms, 2) fast analyses on high performance computing centers (HPCs) using modern parallelization techniques, and 3) development of analysis infrastructures based on optimization pipelines and new data formats, which can be efficiently interfaced to machine learning utilities.

One study, Šimko et al. explored a novel approach for experimental HEP data analyses based on a declarative rather than imperative paradigm to describe the analysis computational workflow. In this study, the analysis process is structured in the form of a Directed Acyclic Graph, with graph vertices representing a computation unit with its inputs and outputs, and graph edges describing the interconnections of different computational steps. The REANA analysis platform featured here allows one to express the computational analysis steps based on the declarative paradigm implemented via the Yadage workflow specification language. REANA parses the analysis workflow and dispatches its computational steps to various supported computing backends (i.e., Kubernetes, HTCondor, Slurm). The approach was tested on two analysis examples from the LHC: 1) reinterpretation of a search for dark matter production in association with a Higgs boson decaying to $b\bar{b}$ in terms of a new physics model, and a derivation of the jet energy corrections in CMS. In the object calibration study, REANA greatly reduced the time and workload of the analysis procedure.

A second study Mosciatti et al., focused on improving the effectiveness of software containers which provide all dependencies needed to run a software workload independently on a host environment, enabling full reproducibility of the workloads and consequently their analysis content and results. Containers are extremely important at the LHC where software environments and operating systems become quickly outdated. One issue is that, the increase in the complexity of analysis software increases the size of container hosting the analysis, making its download and execution unpractical and slow. The study explored a new method to speed up the container download and execution by combining the so-called lazy pulling method, which allows to download image data as needed, and cache on demand in the CERN Virtual Machine File system (CVMFS). This method, which is implemented in the Kubernetes platform, reduces the container start time by at least a factor two compared to the standard download utilities.

Another study Unel et al., focused on techniques to effectively express the physics content of HEP data analyses. It features a domain-specific, declarative language called Analysis Description Language (ADL) that describes the contents of an analysis in a standard and unambiguous way, independent of any computing framework. ADL can be used by experimental and phenomenological communities to facilitate the abstraction, design, visualization, validation, combination, reproduction, interpretation, overall communication and long-term preservation of analysis contents. In ADL, analyses are written in plain text files consisting of blocks separating object, variable and event selection definitions. Blocks have a keyword-expression structure, where keywords specify analysis concepts and operations. Syntax includes mathematical and logical operations, comparison and optimisation operators, reducers, four-vector algebra and commonly used functions. Analyses written in ADL can be executed on event data either via converting ADL to a compilable code by a transpiler, or via direct runtime interpretation. This study described recent advancements in ADL and in particular CutLang, a runtime interpreter of ADL.

Another study Diblen et al., presents works on building a versatile vizualization tool of scientific data that allows analysis of data over the web. The study features SPOT, a tool that can be interfaced to scientific repositories such as Zenodo and HEPData. The main aim of the tools presented here is the quick and convenient re-analysis and comparisons of phenomenological data, which may lead to finding new correlations in published data. SPOT can create 1-3 dimensional visualisations by creating histograms of high-dimensional datasets, and perform interpolation to generalize data. The functionality was demonstrated and benchmarked using different examples that range from the visualization of the parameters of a 19-dimensional supersymmetry model dataset versus their exclusion status by the ATLAS experiment or their consistency with the galactic center excess model, and visualization of simulated LHC data and of the Fermi FL8Y Point Source Catalogue.

Machine learning methods are becoming more central in HEP data analysis due to their extensive discrimination and regression powers. Another study Adams, presented a new technique to remove background particles in events collected by the SBND detector, the near detector in the Fermilab Short-Baseline Neutrino Program, by applying deep learning on full detector images. The paper explored techniques to discriminate the origin of recorded activity to be from cosmic particles, neutrino interactions or background-noise at a single pixel level using deep convolutional neural networks on simulated data from the SBND LArTPC detector. Various DNNs were designed and different metrics were considered to optimize the prediction accuracy. The technique developed is applicable to other LArTPC detectors running at surface level, such as MicroBooNE, ICARUS and ProtoDUNE.

